# Study on Photodeformation of Solvent Resistance in Hydrogen-Bonded Cross-Linked Main-Chain Azobenzene Films

**DOI:** 10.3390/molecules30102106

**Published:** 2025-05-09

**Authors:** Zhaoyang Zhang, Shengkui Ma, Jianfeng Gao

**Affiliations:** 1 School of Chemistry and Chemical Engineering, North University of China, Taiyuan 030051, China; zhaoyangzhang98@163.com; 2 State Key Laboratory of Medicinal Chemical Biology, Key Laboratory of Functional Polymer Materials (Ministry of Education), Tianjin Key Laboratory of Functional Polymer Materials, College of Chemistry Nankai University, Tianjin 300071, China; mashengkui@yeah.net

**Keywords:** azobenzene, main-chain semi-crystalline polymers, physically cross-linked, photodeformation, solvent resistance

## Abstract

Hydrogen-bonded cross-linked main chain azobenzene (azo) photoactive polymers have broad application prospects in flexible actuators, optical actuators, and other fields. Most of the research on this kind of photoresponsive material is mainly focused on air, and exploration in solvents remains underexplored. In this paper, azobenzene polyamide ester semicrystalline polymer (PEA-6T) with hydrogen-bond cross-linking was synthesized by Michael addition polymerization. The uniaxially oriented polymer film with high orientation (48.85%) and fast response (5 s under UV light and 55 s under visible light) was obtained by a simple solution casting/mechanical stretching method. Compared with PEA-2T and PEA-4T, PEA-6T exhibits enhanced mechanical properties (elastic modulus increased by 17.4%; yield strength increased by 34.1%; breaking strength increased by 75.4%; elongation at break increased by 33.8%; toughness increased by 101.3%; photoinduced stress increased by 43.5%) and reduced light response time (decreased by 58.3% in ultraviolet light and 50% in visible light) due to the elongation of the compliant chain length. The thin PEA-6T film exhibited light-induced deformation not only in air but also in polar solvents such as water, methanol, ethanol, butanol, and saline solutions (e.g., normal saline, 0.9 wt% NaCl, and simulated seawater, 3.5 wt% NaCl). In addition, polarizing optical microscope (POM) observations showed that the brightness and texture direction of the films remained stable (Δ_Brightness_ < 5%), the light response time was consistent (6 s under UV light, 65 s under visible light), the light-induced stress retention rate was 95%, and the films exhibited good solvent resistance. This study bridges the research gap in azobenzene photoresponsive materials in solvent environments, and the material shows potential for applications in marine equipment coatings or biomedical actuators.

## 1. Introduction

Photodeformable azo polymers, as a core system in the field of intelligent responsive materials, have demonstrated irreplaceable application value in optical driven microdevices, biomimetic actuators, and adaptive optics systems [1,2]. The core of these materials lies in the photoisomerization behavior of the azobenzene group: under specific wavelength illumination, the azobenzene group can undergo reversible transitions between cis and trans configurations, triggering geometric changes at the molecular scale and rearrangement of dipole moments [3,4]. However, the light responsiveness of the azobenzene group alone is insufficient to achieve macroscopic deformation of the material; the key is how to effectively transmit microscopic molecular motion to the macroscopic scale through rational polymer structural design [5,6]. In this context, the introduction of cross-linked structures has become a core strategy for regulating light deformation performance, as it not only imparts excellent mechanical stability to the material but also achieves efficient conversion of microscopic isomerization behavior to macroscopic deformation by fixing the orientation of the azobenzene groups and accumulating photo-induced stress [7].

At present, researchers have developed two main strategies for constructing photo-induced deformation polymer networks: covalent crosslinking and dynamic non-covalent crosslinking. Although traditional covalent crosslinking systems (such as epoxy resin crosslinking [8] and free radical polymerization crosslinking [9]) construct three-dimensional networks through high-strength covalent bonds (bond energies of 200–400 kJ/mol), endowing materials with excellent mechanical properties and thermal stability, permanent chemical bonds form rigid networks. However, irreversible crosslinking results in the inability of materials to undergo thermoforming or solvent reprocessing after curing, severely restricting device reconstruction and resource cycling. Although dynamic covalent crosslinking [10,11,12,13,14] (such as Diels–Alder reversible bonds [15,16] and disulfide bond exchange [8,17]) endows materials with a certain degree of reparability, it requires complex molecular design and precise synthesis control. In contrast, systems based on dynamic non-covalent crosslinking, such as hydrogen bonding, electrostatic interactions [18,19,20,21,22], π-π interactions [23,24], and self-assembly induced physical crosslinking, can form stable networks through reversible interactions while retaining the material’s thermoplastic processing properties, providing new ideas for developing reproducible photo deformable materials. The semi-crystalline polymer crosslinked by hydrogen bonding constructs a dynamic crosslinked structure through non-covalent interactions such as hydrogen bonding and crystalline networks, which not only preserves the material’s photoresponsiveness but also enhances the polymer’s processability and cycling stability. Zhou [25] used Michael addition polymerization to prepare a semi-crystalline polymer crosslinked by hydrogen bonds in the main chain. The hydrogen bonds induced by amide units and the crystalline domains in the prepared films and fibers endow them with dynamic but stable crosslinking points, making them easily programmable to transform into various three-dimensional (3D) shapes at room temperature. Woojin Lee [26] prepared a dual dynamic supramolecular network by using semi-crystalline star-shaped poly (ε-caprolactone) s (USPs) functionalized with urea pyrimidinone (UPy) end effectors. The appropriate arm length balance of USP can provide a mechanically rigid semi-crystalline supra-molecular network. Its reversible dual dynamic properties, stemming from the reassociation of UPy quadruple hydrogen bonds and the recovery of crystal physical bonds during healing, endow it with efficient healing properties. In addition, by doping polydopamine (PDA) nanoparticles into the main chain of azobenzene semi-crystalline poly (ester amide) (PEAs), a photoactuator with UV-NIR dual photoresponsiveness, room temperature 3D shape reprogramming and reprocessing, and photothermal healing properties is manufactured through hydrogen bonding between PEA and the polymer [27]. In recent years, non-covalently crosslinked photo deformable azo polymers have shown two major development directions: side-chain [28] and main-chain [29]. Side chain systems (such as polyacrylate grafted azobenzene [30,31,32,33,34]) can achieve millisecond-level response speed by connecting photoactive units through flexible spacers (such as -(CH_2_)_6_-) [35], but their applications are still limited [36]. The main chain design directly embeds azo monomers into the polymer main chain (such as polyamide azo-benzene alternating copolymer [37]) to form continuous stress transfer channels. Combined with π-π stacking between the main chains [23] and hydrogen bond arrays, a hierarchical network is constructed, which improves the optical strain efficiency by 2–3 times [38] and the cycling stability exceeds 100 times without attenuation [39]. This structural advantage has attracted much attention in fields such as optically driven pumps that require continuous mechanical output.

In addition, the strength of hydrogen bonds between the main chains can be flexibly regulated through the design of functional groups, such as polymers containing both ester and secondary amine groups (i.e., poly (ester secondary amine) or PesAs) [40], polymers containing both ester and amide groups in the skeleton (such as poly (ester amide) or PEAs) [25,37,41], and polymers containing both ester and urea groups [42] in the skeleton and external environment. The regulation of these microstructures provides possibilities for the multifunctionality of materials. Therefore, this structural advantage makes the main chain type material significantly superior to traditional side chain systems in terms of cycling stability and deformation amplitude. At present, research on hydrogen-bonded crosslinked azobenzene materials mainly focuses on air, with only a small portion being tested in water [27,43] and non-polar reagents [44].

**Scheme 1:**
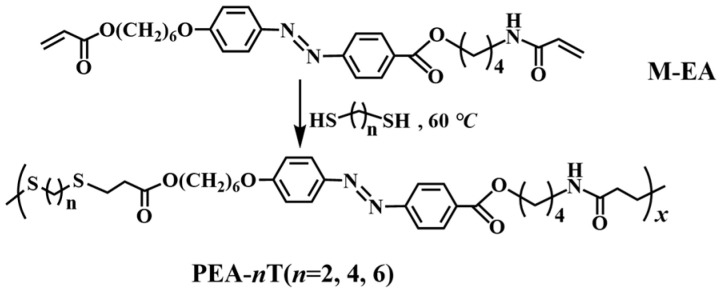
Synthetic routes and chemical structures of the main-chain azo semi-crystalline PEA-*n*T (prepared via the Michael addition polymerization of M-EA with different chain lengths of dithiol (i.e., HS(CH_2_)_n_SH (*n* = 2, 4, 6)).

Although progress has been made in crosslinking azo polymers with hydrogen bonds in the main chain, their long-term stability in solvent environments remains a key but not yet fully explored challenge. The existing research mainly focuses on the photo deformation performance in air or short-term solvent exposure (<24 h), and there is little systematic study on solvent resistance, especially the ability to maintain photo actuation efficiency after prolonged immersion (several days to weeks). This gap severely limits their application in biomedical devices that require physiological saline compatibility or industrial actuators that operate in organic media. To address this issue, we synthesized a dynamic and robust network system through Michael addition polymerization between azobenzene monomer with acrylic ester on one end and acrylamide on the other end, and dithiol with different chain lengths (*n* = 2, 4, 6), as shown in Scheme 1 below.

The aims of this work include the following three aspects: (i) The mechanical properties and light response time as a function of flexible chain length in the main-chain polymer were explored, and a newly designed three-dimensional programmable and recyclable driver at room temperature was developed. (ii) The regularity of light deformation time and bending angle in uniaxially oriented azo polymer films was systematically evaluated under immersion in water (25, 30, 40, and 50 °C), NaCl aqueous solutions (0.9 wt% and 3.5 wt% at 25 and 50 °C), and common alcohol solutions (methanol, ethanol, and *N*-butanol). (iii) The persistent solvent resistance of photoinduced deformation in main-chain azo polymers across different polar solvents was investigated. Firstly, solvent–polymer interactions were analyzed via Fourier transform infrared spectroscopy to characterize the absorption changes in functional groups in polymer films after immersion. Additionally, time-dependent solvent resistance tests (soaking for up to 30 days) were conducted to characterize the maximum light deformation angle, variations in photoinduced stress, orientation changes, and quantitative POM brightness alterations of the films under different soaking durations.

## 2. Results and Discussion

### 2.1. Synthesis and Characterization of the Main-Chain Azo Semi-Crystalline Poly(ester-amide) S (PEAs)

We prepared acrylate-amido azo monomer, and the Michael addition polymerization of M-EA with three dithiols (including 1,2-ethanedithiol, 1,4-butane-dithiol, and 1,6-hexane-dithiol) was carried out in THF at 60 °C (i.e., M-EA, Scheme 1). The polymerization system was homogeneous during the reaction. Orange PEAs with different flexible spacers (PEA-*n*T, *n* = 2, 4, 6) were obtained in high yield (90–92%) within 3 h of polymerization (Table 1). TLC verification confirmed no monomer residue in the polymer products.

**Figure 1:**
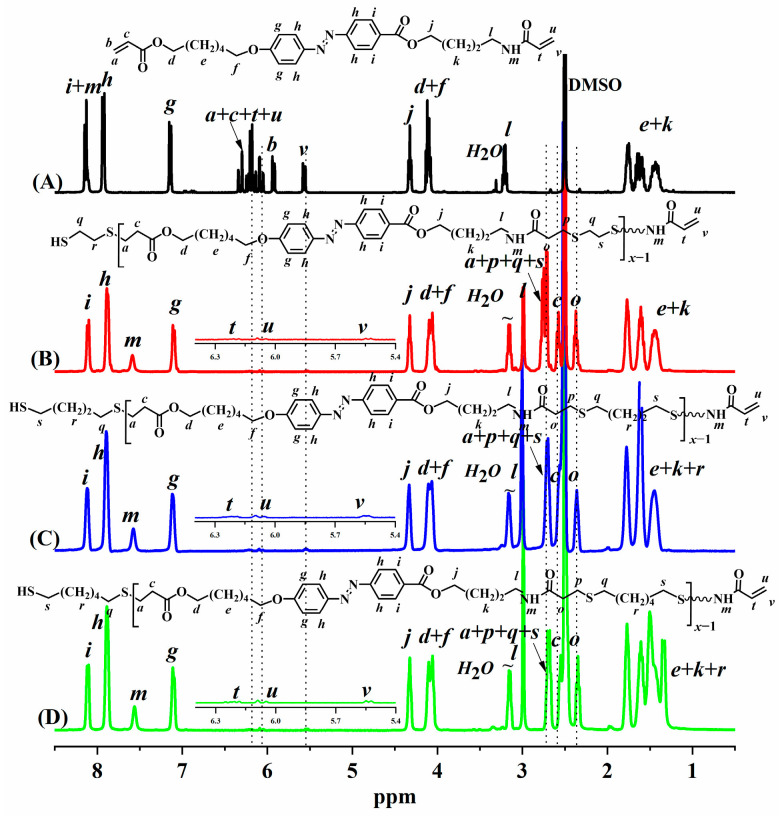
^1^H NMR spectra of M-EA (**A**), PEA-2T (**B**), PEA-4T (**C**), PEA-6T (**D**), and in DMSO under 80 °C.

The azo monomer M-EA and azo polymers PEA-*n*T (Scheme S1) were characterized by ^1^H NMR spectroscopy (Figure 1). As shown in Figure 1, the proton signal of the acrylate-amide group in M-EA is barely detectable in the ^1^H NMR spectrum of PEA-6. The number-average molecular weight (*M_n_*_, *NMR*_) was calculated using the formula: *M_n_*_, *NMR*_ = *x* ∗ *M_repeat unit_* = [(S*g*/S*u*)/2] ∗ *M_repeat unit_*. 

In the formula, “*x*” denotes the number of repeating units; *M_repeat unit_* is the molecular weight of the repeating units; S*u* and S*g* correspond to the peak integrals at 5.86–5.92 ppm (proton “*u*” in the terminal acrylate group) and the peak value of 7.10 ppm (proton “*g*” in the benzene ring), respectively. The number average molecular weight (*M_n_*) is obtained.

**Figure 2:**
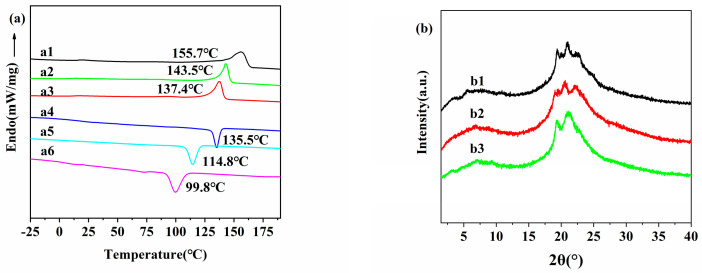
(**a**) The DSC thermogram of PEA-*n*T (*n* = 2 (a1, a4), *n* = 4 (a2, a5) and *n* = 6 (a3, a6)) from the second heating scan (a1–a3) and first cooling scan (a4–a6) (±10 °C min^−1^), while second heating scans are a1, a2 and a3. (**b**) The XRD spectra of PEA-*n*T (*n* = 2 (b1), *n* = 4 (b2) and *n* = 6 (b3)).

The thermal stability and phase transition behaviors of PEA-*n*T (*n* = 2, 4, 6) were then characterized by TGA, DSC, POM, and XRD. To directly assess the thermal stability, the TGA was performed. The thermal decomposition temperature (T_d_) of the polymer was determined by monitoring mass changes with temperature during heating, which reflects the material’s high-temperature stability. TGA analyses revealed that PEAs exhibit high thermal stability, with 5% weight loss temperatures of 288, 292, and 294 °C for n = 2, 4, and 6, respectively (Table 1). DSC was used to quantitatively analyze phase transition behavior and thermodynamic properties, including glass transition temperature (T_g_) and melting temperature (T_m_), which were determined by measuring endothermic or exothermic peaks during heating/cooling cycles. The DSC thermogram of PEAs showed a relatively weak glass transition step (only during the second heating scan) and a phase transition peak (during the first cooling and second heating scans) (Figure 2a). Their glass transition temperatures (T_g_) were 16.4, 12.6, and 8.3 °C for *n* = 2, 4, and 6, respectively, all lower than room temperature (25 °C). The DSC results indicated that in the PEA-*n*T polymers, both the first cooling and second heating curves exhibited a glass transition temperature. T_g_ is the temperature at which the transition from a glassy to a highly elastic state occurs. Glass transition is an inherent property of amorphous polymers and a macroscopic manifestation of changes in polymer molecular motion. It directly affects the material’s performance and processability. POM (polarizing optical microscopy) utilizes birefringence to visualize the morphology, size, and texture of crystalline regions in polymers. POM images of PEA-*n*T are shown in Figure S1. XRD was used to quantitatively calculate crystallinity and correlate phase transition behavior with crystal structure. The XRD spectra of PEA-*n*T exhibited sharp diffraction peaks in the wide-angle region (2*θ* ≈ 21.5°), suggesting a highly ordered semi-crystalline phase (Figure 2a). Assuming that a purely crystalline sample has a typical ΔHm ≈ 150−300 J/g, the crystalline degree of PEAs was derived to be around 10–20% [45] (Figure 2b).

**Figure 3:**
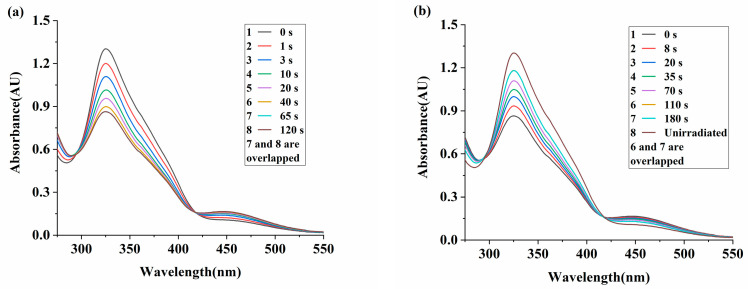
Photoresponsivity of the PEA-6T thin film in the UV (**a**) and visible light (**b**) UV–vis spectral changes in dependence of time for the thin PEA-6T film (cast on the quartz glass plate, film thickness: 740 nm) upon its exposure to 365 nm of UV light (40 mW cm^−2^) and upon irradiating the polymer film at the photostationary state with visible light (λ > 510 nm, 30 mW cm*^−^*^2^) at 25 °C.

The photoresponsivity of a representative azo polymer (PEA-6T) thin film was investigated. The photoresponse of PEA-6T thin films is shown in Figure 3a,b. UV–vis spectral changes were observed under ultraviolet (λ = 365 nm, 40 mW cm^−2^) and visible light (λ > 510 nm, 30 mW cm^−2^) irradiation. There is a strong absorption peak at 325 nm and a weak absorption peak near 450 nm, corresponding to the π-π* electron transition (trans isomer) and n-π* electron transition (cis isomer) of the nitrogen–nitrogen double bond (-N=N-) in the azobenzene group, respectively.

As shown in Figure 3a, under ultraviolet light irradiation, the intensity of the π-π* electron transition absorption peak near 325 nm decreases significantly, while the intensity of the n-π* electron transition peak near 450 nm increases slightly. After 65 s of ultraviolet irradiation, the PEA-6T film reaches a photostable state. The UV–vis spectrum shows two equal absorption points, indicating that the film undergoes only cis-trans isomerization of the azobenzene group under UV irradiation, without side reactions [46]. Subsequently, the aforementioned photostable film was exposed to visible light (λ > 510 nm, 30 mW cm^−2^) to investigate its recovery behavior. Figure 3b shows that under visible light irradiation, the absorption peak intensity at 325 nm increases, while that near 450 nm decreases slightly, indicating the gradual recovery of azobenzene groups from cis to trans isomers. After 110 s of visible light irradiation, the PEA-6T film reached a photostable state. According to the absorbance values, approximately 89% of the cis isomer converts to the trans isomer, but the film cannot fully recover its initial state before UV irradiation, as previously reported [47,48]. The maximum absorption peak of the azobenzene polymer in the solid film state exhibits a blue shift, from 365 nm in solution to 325 nm in the film. This is attributed to restricted azobenzene group mobility in the solid film state and the formation of H-aggregates [49]. In addition, as shown in Figure S2, ten UV–visible light irradiation cycles were performed on the thin film. Similarly, azo polymer PEA-2T and PEA-4T thin films exhibited the same photoresponsivity, as shown in Figures S3 and S4.

### 2.2. Mechanical and Photomechanical Properties of the Uniaxially Oriented Azo Films in Air

**Figure 4:**
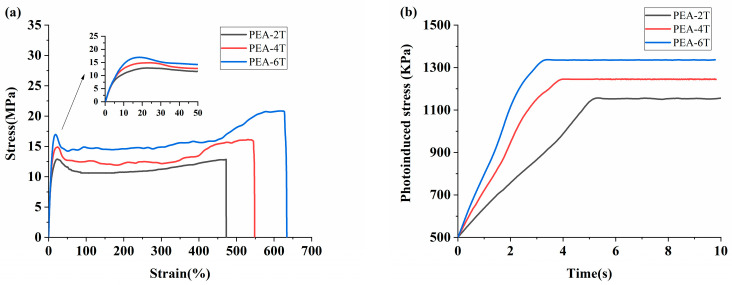
(**a**) Stress–strain curves of unoriented main-chain azobenzene polymer PEA-*n*T films and photoinduced stress of uniaxially oriented azobenzene polymer PEA-*n*T films (a strain of 235%) were measured at 25 °C at a stretching rate of 10 mm min^−1^. (**b**) The photoinduced stress of azobenzene PEA-*n*T (*n* = 2, 4, 6) stretched films with time under ultraviolet irradiation (λ = 365 nm, 40 mW cm^−2^) at 25 °C was investigated.

The main chain azobenzene polymer films were first prepared by the solution casting method, and the mechanical properties of the unstretched films were studied (Figure 4 and Table 2). The lengths and widths of the uniaxially oriented azobenzene polymer films were 10 mm and 2 mm, respectively, and the thicknesses of PEA-2T, PEA-4T and PEA-6T were 63, 63, and 64 μm, respectively. And the photoinduced stress was measured by the stretched PEA-*n*T films with a strain of 235%. An external force of 500 kPa was applied to the films. The dimensions (length ∗ width ∗ thickness) of the azobenzene polymer stretched films are PEA-2T (10 mm ∗ 1 mm ∗ 37 μm), PEA-4T (10 mm ∗ 1 mm ∗ 38 μm), and PEA-6T (10 mm ∗ 1 mm ∗ 37 μm).

The uniaxially oriented PEA-*n*T films showed homogeneous azo mesogen alignment along the film axes, as revealed by a sharp contrast inversion every 45° upon rotating the samples with respect to the analyzer under crossed POM observation, as shown in Figure S5.

The photoinduced stress curves of uniaxially oriented PEA-*n*T (*n* = 2, 4, 6) were recorded. To ensure test accuracy, the two ends of each film were clamped to secure the film’s position, and an initial stress of 500 kPa was applied to keep its length constant. Subsequently, time-dependent photo-stress values of the films under ultraviolet light (λ = 365 nm, 40 mW cm^−2^) were recorded. For PEA-2T, PEA-4T, and PEA-6T, the values were 582.7 ± 43.1, 722.5 ± 48.6, and 835.9 ± 48.2 kPa, respectively, at ambient temperature. The photoinduced stress of the films increased with the elongation of the flexible spacer length, and the degree of orientation increased accordingly.

By measuring small-angle X-ray scattering (SAXS) (Figures S6 and S7), the degree of orientation in PEA-*n*T for *n* = 2, 4, and 6 was determined to be 46.41%, 47.16%, and 48.85%, respectively.

**Figure 5:**
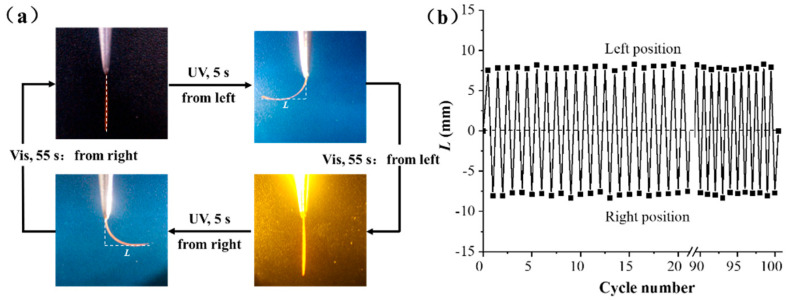
(**a**) Photo of photo-induced bending/non-bending of azobenzene polymer PEA-6T stretch film under 365 nm ultraviolet light (40 mW cm^−2^) and visible light (>510 nm, 30 mW cm^−2^) irradiation at room temperature. (**b**) The reversible deformation of the PEA-6T film characterized by tracing the bent distance from its straight state at room temperature.

For air-induced deformation testing, we obtained a PEA-6T polymer film with a thickness of ~63 μm (measured by metallographic microscope) and a strain of 235%. The dimensions were 12 mm ∗ 1 mm ∗ 37 μm, and a 2 mm segment of the film was clamped with a flat clip. The uniaxially oriented PEA-6T film bent to the left toward the UV light source (λ = 365 nm, 40 mW cm^−2^), reaching maximum bending within 5 s. The bent film returned to its initial straightness after irradiation and was irradiated with visible light (λ > 510 nm, 30 mW cm^−2^) for 55 s, as shown in Figure 5. Thus, the maximum response time for UV/visible light, bending/non-bending duration, and bending amplitude/angle were obtained. Results consistent with the photoinduced stress measurements were obtained through testing.

In addition, the uniaxially oriented PEA-6T film exhibits excellent fatigue resistance through its reversible deformation over 100 cycles, with nearly constant bending/extension times and bending amplitudes, as shown in Table 3. Similar to the uniaxially oriented PEA-6T film, PEA-2T (Figure S8) and PEA-4T (Figure S9) stretched films (with a strain of 235%) also exhibit significant and reversible photo-induced bending and stretching. Due to its excellent reversibility and fast photoresponsiveness, PEA-6T was selected as the experimental material to study the adaptability of azo main-chain polymer films in different polar solvents and their ability to resist solvents over a prolonged period.

### 2.3. Room Temperature Three-Dimensional Shape Programmability and Recyclability of Uniaxial Oriented PEA-6T Films

In addition, we investigated the three-dimensional shape programmability of uniaxial oriented PEA-6T films. Similar to the main-chain azo polymers reported earlier [50], stable 3D photo-drivers were easily obtained by reprogramming all oriented films prepared from main-chain azo LCPs strained at room temperature, as shown in Figure S8.

Next, the high recyclability and reprocessability of uniaxially oriented films were demonstrated. For PEA-6T films, the original film was dissolved in hexafluoro isopropanol, the solvent was evaporated to dryness, and then the residue was vacuum-dried (all steps were carried out at room temperature). This simple room-temperature recovery capability of PEA-6T-based photo-actuators offers a clear advantage over photo-actuators that can only be recovered by heating or reversible chemical reactions, primarily because the polymer remains intact during this gentle recovery process. In addition, the recovered PEA-6T can be further reprocessed into uniaxially oriented films by solution pouring/mechanical drawing and can display reversible photoinduced bending/non-bending with high fatigue resistance (over 100 cycles), exactly like the original film, as shown in Figure S10.

The recycling method for PEA-6T thin films involved dissolving the films in DCM, followed by evaporation of the solvent to dryness and drying under vacuum until a constant weight was achieved (all procedures were conducted at room temperature), as shown in Figure S11. The recovered product is then characterized using ^1^H NMR, as shown in Figure S12.

### 2.4. Photomechanical Properties and Solvent Resistance of Uniaxially Oriented Films of Main-Chain Azo PEAs Under Common Hydrogen Bonding Solvents

The presence of hydrogen bonding and crystalline domains in polymer chains endows main-chain physically cross-linked polymers with photodeformation, providing an intrinsic driving force for their micro to macro photo-induced bending [51]. Most researchers focus on studying different light intensities and temperatures [8,50]; however, few investigate the adaptability of photodeformation in air or explore long-term solvent resistance in polar environments [52]. Therefore, it is extremely important to systematically evaluate adaptability in different solvents (such as water, methanol, ethanol, butanol, and saline solutions), including initial photodeformation time and bending angle.

Firstly, a uniaxially oriented PEA-6T film (235% strain) with dimensions of 12 mm ∗ 1 mm ∗ 36 μm (length ∗ width ∗ thickness) was obtained via solution casting/mechanical stretching, with a 2 mm segment clamped by tweezers. We tested the UV–visible light cycling of the polymers in water (25 °C, 30 °C, 40 °C, 50 °C), saline solutions of different concentrations (0.9 wt%, 3.5 wt%) at 25 °C and 50 °C, methanol, ethanol, and butanol. The maximum bending distance (L) and the time required for light deformation to reach maximum bending (t) were recorded using a mobile phone, as shown in Table 3.

In order to study the relationship between solvent polarity and photodeformation time, we first analyzed various parameters of the following solvents. Uniaxially oriented PEA-6T film (235% strain) was tested for contact angle in different solvents, as shown in Figure S13 and Table S1. The water contact angle was relatively large, indicating weak wettability, which suggested partial compatibility between the solvent and the polymer surface but incomplete penetration. However, the bending and non-bending rates of the uniaxially oriented PEA-6T film in water were slightly slower than in air, likely due to the greater viscous resistance of water to the film. Therefore, measurements of the infrared spectra of uniaxially oriented PEA-6T film before and after light deformation in water showed that the intensity ratio of the sur-rounding bands was basically the same, ranging from 3306 cm^−1^ to 3377 cm^−1^ (which originated from hydrogen bonds and “free” N-H extending from amide units, and the band near 1639 cm^−1^ was approximately 1670 cm^−1^ (corresponding to hydrogen bonds and “free” C=O extending from amide units, respectively), revealing the hydrogen bonds between amide units in the PEA-6T film caused by water, as shown in Figure S14.

The moderate wettability of methanol and ethanol, as indicated by contact angles, allows solvent molecules to partially swell the material surface, reducing the energy barrier for chain segment movement while maintaining the overall stability of the hydrogen-bond cross-linked network. Therefore, the film could be cycled up to 100 times in methanol/ethanol, with a lower degree of change compared to water. Dynamic UV light overlay (every 0.5 s) and visible light overlay (every 5 s) were recorded, as shown in Figure S15. Although the contact angle of butanol is close to those of methanol and ethanol, the polymer’s photodeformation experienced strong viscous resistance, twice that of water, and its Hansen parameter was the smallest, reducing its photodeformation speed by ~33.3% compared to ethanol.

Mechanism analysis revealed faster photodeformation in physiological saline (0.9% NaCl) than in pure water, while high-concentration saline (3.5% NaCl) showed slower speeds (Table 3, Entries 8 and 9). The “ion shielding effect” of 0.9% NaCl refers to partial shielding of the polarity of amide bonds (-CONH-) in polymers by low-concentration salt ions (Na^+^/Cl^−^), weakening intramolecular/intermolecular hydrogen-bond cross-linking and reducing segment motion resistance. Dynamic enhancement of hydrogen-bond networks facilitates segment rearrangement driven by azobenzene photoisomerization, accelerating photodeformation, while strong hydration of high-concentration salt ions reduces free water, weakening solvent–amide hydrogen bond competition. Meanwhile, excessive salt ions cause polymer chains to aggregate through electrostatic attraction (similar to the “salting out effect”), leading to network rigidity and suppressing photoresponsive deformation.

We tested the photoresponse of pure water under different temperature gradients. The primary driver of accelerated photodeformation is the activation of chain segment motion. Increasing temperature (25–40 °C) provides thermal energy, reduces the potential barrier for polymer chain segment motion, and promotes the translation of local configuration changes, induced by photo-induced cis-trans isomerization (trans-cis) of azobenzene groups, into macroscopic deformation. Dynamic enhancement of hydrogen bonds involves thermal weakening of amide–amide hydrogen bonding, which induces “temporary softening” of the crosslinked network, enabling segment rearrangement and accelerating deformation response. Enhanced solvent permeability arises from temperature-induced viscosity reduction, promoting water/saltwater infiltration into the material surface, which enhances swelling, free volume, and deformation capacity.

While increasing temperature accelerates light response, it also disrupts hydrogen bonds, since hydrogen bond energy is typically 25–40 kJ/mol, leading to loss of weak intermolecular interactions at excessively high temperatures. Notably, the above-mentioned uniaxially oriented PEA-6T film exhibits a larger bending angle than previously reported (Table S4). It was not only oriented and stretched at room temperature but also maintained good photoinduced deformation and reversible cycling in various solvents. This is also the significance of developing and exploring the photoresponsivity of such polymer films in different solvents.

Photoresponsive drivers in air exhibit long-lasting and stable responses. The solvent resistance of the prepared main-chain uniaxially oriented PEA-6T polymer in photodeformation tests was evaluated as follows.

To investigate the persistent solvent resistance of physically crosslinked photodeformable main-chain azo polymers in different polar solvents, we conducted long-term soaking tests. Firstly, the films were placed in corresponding test solvents for 7, 14, and 30 days, and their maximum bending angles were measured after each soaking duration. (Note: Soaking time for 7 h means directly placing the uniaxially oriented polymer films into a solvent to test the results of 100 cycles, as shown in Table 3.)

**Figure 6:**
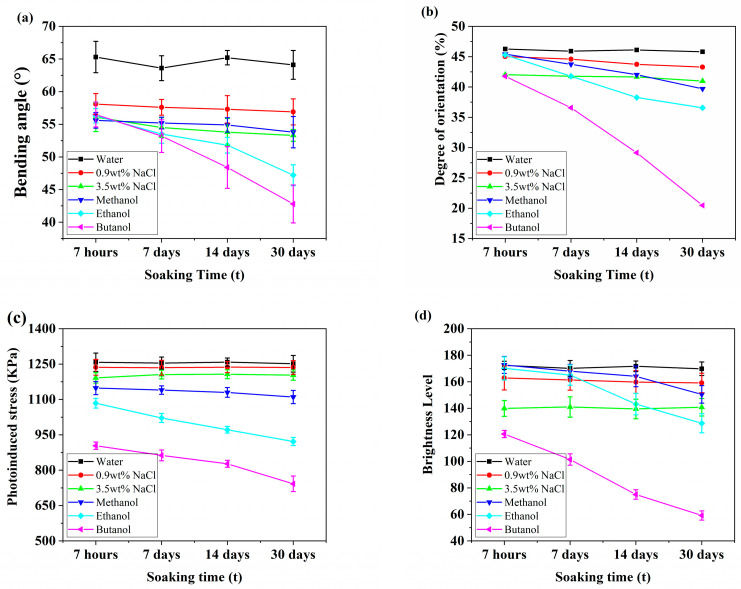
(**a**) Maximum bending angle in different solvents at different soaking times. (**b**) Orientation of polymer films in different solvents at different soaking times. (**c**) Photo-induced stress in different solvents at different soaking times. (**d**) Brightness level of POM in different solvents at different soaking times. (Soaking time for 7 h means directly placing the uniaxially oriented polymer films into a solvent to test the results of 100 cycles, as shown in Table 3).

As shown in Figure S16, the maximum bending angle varies with the immersion time of the films in solvents. Statistical results, including those in Figure 6a and Table S3, are presented below. Notably, consistent with previous tests, the maximum bending angle in water (64.5 ± 0.8°) was greater than in other solvents and remained stable over time. However, the maximum bending angle fluctuated more significantly in water than in saline solutions over time. The maximum bending angle in saltwater solutions of different concentrations remained stable, likely due to dynamic hydrogen bond equilibrium between ions and the main-chain azobenzene polymer films. It is worth noting that it can be clearly seen that the maximum bending angle of the polymer film in the alcohol solvents decreases to varying degrees with the prolongation of soaking time. After soaking in methanol for 30 days, the polymer film exhibited significant changes. The polymer film showed significant changes after soaking in ethanol and butanol for 14 days and 7 h, respectively. To investigate the reasons, we tested the changes in orientation and photo-induced stress of the polymer film soaked for different times, as shown in Figure 6b,c, and Table S3. To ensure test accuracy, the two ends of the film were clamped to secure its position, and an initial stress of 500 kPa was applied to maintain constant length. Subsequently, the time-dependent photo-stress of the film under ultraviolet light (λ = 365 nm, 40 mW cm^−2^) was recorded and shown in Figure 6c. Photoinduced stress results showed distinct magnitudes and trends for polymer films soaked in different solvents for different times. In water, the larger contact angle resisted infiltration, thus minimizing photoinduced stress changes. In NaCl aqueous solutions, slightly smaller contact angles caused moderate film damage. Methanol, ethanol, and butanol had similar small contact angles; however, their different solubility parameters caused distinct degrees of film damage.

The orientation degree of saltwater is lower than that of water, which may be attributed to the dynamic equilibrium of hydrogen bonds between ions and the main chain azo-benzene polymer film, and its maximum bending angle remains unchanged; It is worth mentioning that from Figure 6a, the maximum bending angle in saltwater is more stable. Alongside Figure 6a results, analysis of orientation and photoinduced stress in methanol-soaked films revealed minimal damage to hydrogen bonds and film crystallinity. That is, even though orientation changed slightly, the maximum bending angle of uniaxially oriented films remained nearly unchanged (only a 3.3% reduction). However, the solvent resistance of the polymer films in ethanol/butanol was poor. From day 14 onward, orientation in ethanol-soaked films decreased significantly, accompanied by a 16.2% reduction in maximum bending angle. The change in the maximum bending angle of the polymer film after soaking in butanol was significant, indicating severe disruption of hydrogen bonds and crystallinity in the polymer films.

**Figure 7:**
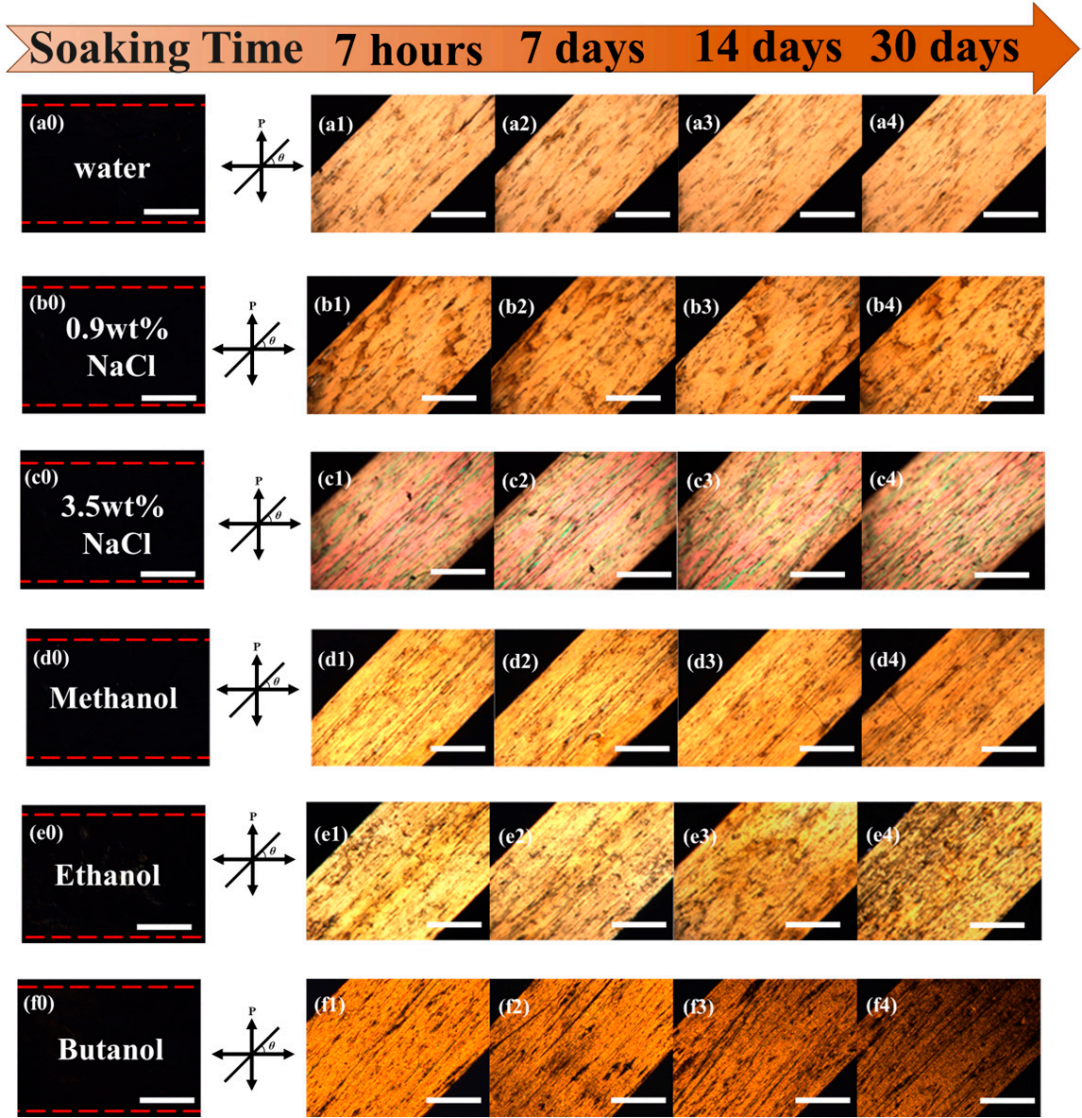
POM of uniaxially oriented azobenzene polymer PEA-6T in different solvents for different soaking times.

Figure 7 shows the Polarizing Optical Microscopy (POM) of polymer films soaked in different solvents for different durations. These tests were conducted at room temperature using polymer films with a strain of 235%. Specifically, the polymer films were observed after immersion in water (a0–a4), 0.9% NaCl aqueous solution (b0–b4), 3.5% NaCl aqueous solution (c0–c4), methanol (d0–d4), ethanol (e0–e4), and butanol (f0–f4). The angles *θ* between these membranes and the analyzer are 0° (a0, b0, c0, d0, e0, f0) and 45° (a1–a4, b1–b4, c1–c4, d1–d4, e1–e4, f1–f4), respectively. Among them, a1–f1 correspond to 7 h, a2–f2 to 7 days, a3–f3 to 14 days, and a4–f4 to 30 days of soaking time, respectively. Microscopic images allowed clear observation of the films’ microstructure and orientation. The scale bar in the images is 500 μm. As soaking time increased, POM images of methanol-soaked (30 days), ethanol-soaked (14 days), and butanol-soaked films showed significant changes consistent with the aforementioned photoinduced stress and orientation results. The results clearly indicate that the solvent affects the overall orientation of the films by influencing their crystalline network. In addition, POM images show that the films expanded to varying degrees in methanol, ethanol, and butanol, consistent with Hansen parameters (Figure S17). Brightness levels in POM images under different soaking times were analyzed using Image J (Image J1.52a; Java1.8.0_112 [64-bit]; 4644 K of 6066 MB(<1%)), as shown in Figure 6d and Table S2.

FTIR spectra were recorded (Figure S18) in the range of 3306–3377 cm^−1^, corresponding to hydrogen-bonded and “free” N-H stretching vibrations of amide units. A band near 1639 cm^−1^ shifted to ~1670 cm^−1^, indicating hydrogen-bonded and “free” C=O stretching vibrations, respectively. These changes revealed alcohol-induced hydrogen bonding between amide units in PEA-6T films. Notably, a strong absorption peak at 1011 cm^−1^ originated from “free” C-O stretching in alcohols. This indicates that solvents can induce local changes in the films’ network by affecting hydrogen bonds, leading to POM darkening and a decrease in orientation and photoinduced stress.

## 3. Materials and Methods

### 3.1. Materials and Reagents

Tetrahydrofuran (THF, Tianjin Chemical Reagent Co., Ltd., Tianjin, China, analytical grade (AR)) was refluxed over sodium and then distilled. Methanol (Tianjin Jiangtian Chemical Technology Co., Ltd., Tianjin, China, AR) and dichloromethane (CH_2_Cl_2_, Tianjin Chemical Reagent Co., Ltd., Tianjin, China, AR) were refluxed over calcium hydride and then distilled. 4-((4-(ω-hydroxyhexyloxy))phenylazo) benzoic acid (H6AzoC) was synthesized according to previously reported procedure [25]. 1,2-Ethanedithiol (Shanghai Macklin Biochemical Co., Ltd., Shanghai, China, 97%), 1,4-butanedithiol (Shanghai Aladdin Biochemical Technology Co., Ltd., Shanghai, China, 97%), 1,6-hexanedithiol (Shanghai Aladdin Biochemical Technology Co., Ltd., Shanghai, China, 98%), 1,8-diazabicyclo[5.4.0]undec-7-ene (DBU, Tianjin Heowns Biochem Technologies, LLC, Tianjin, China, 98%), 1-Ethyl-3-(3-dimethylaminopropyl) carbodiimide hydrochloride (EDCI, Heowns Biochem Technologies, LLC, Tianjin, China, 98%), 4-(dimethylamino)pyridine (DMAP, Merck, Saskatchewan, SK, Canada, AR), diethyl ether (AR), 4-amino-1-butanol (Heowns Biochem Technologies, LLC, Tianjin, China, 97%), acryloyl chloride (Heowns Biochem Technologies, LLC, Tianjin, China, 98%) and all the other chemicals were commercially available and used directly without further purification.

### 3.2. Synthesis of N-(4-Hydroxybutyl) Acrylamide

4-Amino-1-butanol (3.565 g, 40 mmol) was dissolved in 75 mL of dry chloroform solution, and the solution was cooled to 0 °C by an ice water bath. Dissolve acryloyl chloride (1.625 mL, 20 mmol) in 20 mL of dry chloroform solution. The ice water bath was added with a constant pressure drip funnel, and then continued to stir for 4 h under these conditions. After the reaction, 5 times the amount of silica gel was added directly to remove the solvent, and EA: MeOH = 10:1 was used as the eluent. The white solid product was purified by silica gel column chromatography, and the yield was 71%. ^1^H NMR (400 MHz, DMSO-d6): δ (PPM) = 8.07 (s, 1 H-NH-), 6.28 6.24 (dd, 1 H, CH=CH-), 6.12 6.06 (d, 1 H, CH_2_=CH-), 5.64 5.61 (d, 1 H, CH_2_=CH-), 4.54 4.32 (t, 1 H, -OH), 3.27 2.97 (s, 2 H-NH-CH_2_-), 1.53 1.43 (s, 4 H-(CH_2_)_2_-).

### 3.3. Synthesis of M-EA

N-(4-hydroxybutyl) acrylamide (1.43 g, 10.00 mmol), A6AzoC (1.98 g, 5.00 mmol), and DMAP (61.08 mg, 0.50 mmol) were dissolved in 55 mL of dehydrated dichloromethane, and the solution was cooled to 0 °C by an ice water bath. EDCI (1.15 g, 6.00 mmol) was dissolved in 25 mL of dehydrated methylene chloride solution and slowly added to the reaction system under the condition of ice water bath stirring. After the drip is finished, continue to stir under these conditions for 1 h, and then stir at room temperature, 25 °C, for 24 h. After the reaction, it was washed three times with 10 mL 0.01 M hydrochloric acid and then washed three times with saturated salt to remove excess EDCI and reaction catalyst DMAP. Concentrate the filtrate and add silica gel (200–300), 10 g. The liquid mixture EA:PE (*v*:*v*) = 1:1 was used as eluent by dry method and purified by silica gel column chromatography. The orange–yellow solid product was obtained with yield of 62%. UV–Vis (DMAc): λmax/nm (ε/L mol^−1^ cm^−1^) = 362 (26,670). ^1^H NMR (400 MHz, DMSO-d6): δ (PPM) = 8.20 8.07 (m, 3 H, Ar- and H-NH-CO-), 7.99 7.86 (dd, 4 H, Ar-H), 7.21 7.10 (d, 2 H, Ar-H), (6.38 6.02 m, 4 H, CH=CH-COO- and CH=CH-CON-) 5.98 5.88 (dd, 1 H, CH=C-COO), 5.63 5.54 (dd, 1 H, CH=C-CON-), 4.40 4.26 (t, 2 H, Ar-the COO-CH_2_-), 4.18 4.04 (m, 4 H, CH_2_=CH-COOCH_2_- and -CH_2_-O-Ar), 3.27 3.15 (q, 2 H, CH_2_-NH-), (1.85 1.33 m, 12 H, -(CH_2_)_4_- and -(CH_2_)_2_-).

### 3.4. Synthesis of PEA-nT (n = 2, 4, 6)

M-EA (72.38 mg, 0.14 mmol) was added with dried tetrahydrofuran (THF, 2.25 mL) and 1,8-diazecyclic[5,4,0]undecene-7 (DBU, 25 uL, 0.16 mmol) in a 10 mL single-necked round-bottomed flask, stirring well until dissolved. Under the conditions of an ice water bath, argon gas is injected to remove oxygen for 5 min. Next, an equal molar amount of dimercaptan (0.14 mmoL) was added, and argon was continued for 10 min. Subsequently, the reaction bottle was sealed and placed in an oil bath at 60 °C, and the reaction was carried out under dark conditions for 3 h. After the reaction is completed, the reaction liquid is slowly added to the ice methanol, and the precipitate is obtained by centrifugation. After the solid was collected and dissolved in tetrahydrofuran, it was settled in ice methanol again, operation was repeated, and there was no monomer residue on the silicone plate. The centrifuged solids were collected and dried in a vacuum drying oven at 40 °C until their constant weight was obtained. Finally, the orange polymer PET-*n*T (*n* = 2, 4, 6) was obtained.

### 3.5. Preparation of the Thin PEA-nT Film for the Photoresponsivity Study

A thin PEA-6T film was cast from a PEA-6T solution in hexafluoroisopropanol (2.0 mg mL^−1^, 100 μL) on a clean quartz glass plate (12 ∗ 45 mm). After the solvent was evaporated slowly at ambient temperature for 12 h, a transparent light-yellow film was formed on the quartz glass plate. The thickness (l) of the polymer film is estimated to be 740 nm by using the equation l = VC/ρS, where V is the volume of the polymer solution cast on the quartz glass plate, C is the concentration of the polymer solution, ρ is the density of the solid polymer film (ρ is assumed to be 1 g mL^−1^), and S is the surface area of the quartz glass plate. The photoresponsivity of the resulting thin PEA-6T film was studied by first irradiating it with UV light (365 nm, 40 mW cm^−2^) until its photostationary state was reached. The photostationary PEA-6T film was then irradiated with visible light (λ > 510 nm, 30 mW cm^−2^). The UV–vis spectra of the sample were recorded during the above studies. The UV light and visible light were obtained from a high-pressure mercury lamp (USHIO SP-7 from Tokyo, Japan, glass filters were used to obtain the desired light). And thin PEA-2T film and thin PEA-4T film were prepared using the same method as described above.

### 3.6. Preparation of the XRD Characterization for the Azo Polymer Powders

The crystallinity of the main chain azobenzene polymer was characterized by a Rigaku Smart Lab 9 kW (copper target) X-ray diffractometer from Nippon Science Corporation, Tokyo Japan. Test conditions: Tube voltage 40 kV, tube current 150 mA, 2*θ* scanning range 1.5–40°, scanning rate 5°/min.

The sample preparation procedure for measuring XRD spectra of polymer powders includes first heating the polymer to a temperature higher than its anisotropy temperature (+10 °C min^−1^), then cooling it to a temperature 2 °C lower than the exothermic peak temperature in DSC spectra (−10 °C min^−1^) during the first cooling process, and quenching it with liquid nitrogen to maintain its phase structure (all heating and cooling are carried out under a nitrogen atmosphere).

### 3.7. Preparation of Uniaxially Oriented Main-Chain Azo Polymer Films

Uniaxially oriented PEA-6T films were prepared by solution pouring/mechanical stretching method using the method [53]. Take 100 mg of polymer and fill 1 mL with hexafluoro isopropanol. Take 180 uL and place the bottom of the polytetrafluoroethylene mold with length ∗ width ∗ height (15 mm ∗ 10 mm ∗ 3 mm). The top was covered with a glass petri dish, volatilized at room temperature 25 °C for 24 h, and then the polymer film was cut into a length ∗ width ∗ thickness 12 mm ∗ 2 mm ∗ ~63 um. The polymer film was mechanically stretched at room temperature and cut into a length ∗ width ∗ thickness 12 mm ∗ 1 mm ∗ ~36 um (a strain of 235%) film. Used to measure photoinduced stress, photodeformation, and degree of orientation. 

### 3.8. Determination for Degree of Orientation in Uniaxially Oriented Main-Chain Azo Polymer Films

Degree of orientation of the uniaxially oriented PEA-*n*T films was tested by using Xeuss 3.0 UHR from Xenocs S.A.S (1-3 Allée du Nanomètre 38000 Grenoble, France) in full power state with a power supply of 50 kV and 0.6 mA [54,55]. The preparation method of uniaxially oriented PEA nT films was described above [56]. Carefully apply the non-oriented/uniaxially oriented polymer film horizontally onto the sample stage using polyimide tape (a strain of 235%), which has circular holes with a diameter of 2 mm on the sample stage.

## 4. Conclusions

Low T_g_ (<room temperature) and high-temperature (T_d_ > 288 °C) resistant polymers were effectively synthesized via Michael addition polymerization of semi-crystalline main-chain azo polyester amides (PEAs). Uniaxially oriented polymer films physically cross-linked by solution casting/mechanical stretching exhibit good mechanical strength. Within a certain range, the length of flexible polymer chains significantly influences both mechanical strength and photomechanical properties. Elastic modulus ranges from 179.2 to 210.3 MPa, rupture strength from 12.6 to 18.0 MPa, elongation at break from 472.9% to 632.9%, and photoinduced stress from 582.7 to 835.9 kPa.

Photodeformation tests of uniaxially oriented PEA-6T polymer films in different solvents yielded the following conclusions: First, contact angle and viscosity index influence the response speed and stability of optical deformation. When compared to methanol, the photo response speed of butanol, with a higher viscosity index, is reduced by 33%. Second, the polarity constant of the solution directly affects the strength and kinetics of hydrogen bonds: high-polarity solvents (such as water) do not significantly weaken hydrogen bonds, whereas low-polarity solvents (such as ethanol and butanol) may destabilize the cross-linked network, thereby influencing light response rates and deformation reversibility. This correlates with Hansen’s solubility parameters. At room temperature, changes in POM brightness, photoinduced stress, photoresponsiveness, and orientation of uniaxially oriented polymer films soaked in water or brine for 30 days remain below 5%. In contrast, films soaked in methanol for 30 days, ethanol for 14 days, and butanol even after short-term immersion exhibited significant decreases in POM brightness, maximum bending angle, film orientation, and photoinduced stress. Additionally, polymer films swelled to varying degrees in non-polar solvents. Solvent resistance tests indicated that retarded responses primarily stem from the disruption of hydrogen bonds and crystalline domains.

The study of photodeformation in polar reagents is expected to simulate material performance in saline plasma environments, such as biological fluids or industrial corrosion scenarios, thereby laying the foundation for marine equipment coatings or biomedical actuators. Additionally, solvent adaptability research can uncover general design principles for hydrogen-bond cross-linked networks, facilitating cross-medium applications in flexible electronics, environmental sensing, and other fields. Thus, investigating the photodeformation behavior and tolerance of hydrogen-bonded crosslinked azobenzene polymers in various solvents not only bridges the research gap in non-aqueous systems but also provides a theoretical foundation and technical support for developing smart materials with high environmental adaptability.

## Figures and Tables

**Table 1:** Synthetic and characterization data of the main-chain azo semi-crystalline PEAs.

Entry	Sample *^a^*	Yield (%)	Degree of Polymerization (DP) *^b^*	*M*_n, NMR_ (g·mol^−1^) *^c^*	Thermal Transition *T* (°C) *^d^*	ΔH_si_ (J·g^−1^) *^g^*	T_d_ (°C) *^h^*
1	PEA-2T	90	47	28,400	G 16.3 Cr 155.7 I *^e^* I 135.5 Cr 10.5 G *^f^*	31.3 −26.4	288
2	PEA-4T	91	48	30,900	G 12.6 Cr 143.5 I *^e^* I 114.8 Cr 4.5 G *^f^*	29.4 −24.4	292
3	PEA-6T	90	49	32,900	G 8.3 Cr 137.4 I *^e^* I 99.8 Cr 1.4 G *^f^*	28.6 −25.5	294

*^a^* All the main-chain azo polymers were obtained via Michael addition polymerization of the azo monomer and different chain lengths of dithiol with DBU as the catalyst at 60 °C in THF. *^b^* Degree of polymerization calculated by ^1^H NMR. *^c^* The number-average molecular weight determined by ^1^H NMR. *^d^* G = glassy, Cr = crystalline, and I = isotropic. *^e^* DSC second heating scan under nitrogen (10 °C min^−1^). *^f^* DSC first cooling scan under nitrogen (−10 °C min^−1^). *^g^* Enthalpy of the phase transitions. *^h^* The temperature at 5% weight loss of the polymer under nitrogen determined by TGA heating experiment (10 °C min^−1^).

**Table 2:** Mechanical properties of main-chain azobenzene polymer films and photoinduced stress of uniaxially oriented films.

Entry	Sample	Elastic Modulus (MPa)	Yield Strength (MPa)	Rupture Strength (MPa)	Elongation at Break (%)	Toughness (MJ/m^3^)	Photoinduced Stress (kPa)
1	1,2-film	179.2 ± 4.2	12.6 ± 1.1	12.6 ± 0.7	472.9 ± 28.7	53.7 ± 8.4	582.7 ± 43.1
2	1,4-film	197.5 ± 3.2	14.8 ± 0.9	16.1 ± 1.1	547.6 ± 22.7	72.7 ± 5.2	722.5 ± 48.6
3	1,6-film	210.3 ± 4.0	16.9 ± 1.1	22.1 ± 2.1	632.9 ± 15.4	108.1 ± 2.3	835.9 ± 48.2

**Table 3:** Light-induced bending/non-bending of uniaxially oriented azobenzene polymer PEA-*n*T films under different environments and temperatures.

Entry	Sample	Environment	Temperature (°C)	Induced Bending Time (s) *^a^*		Bending Amplitude L (mm) *^b^*	Cycling Number (times) *^c^*
				UV	Vis		
1	PEA-2T	Air	25	12	110	6.8 ± 0.2	>100
2	PEA-4T	Air	25	10	90	7.0 ± 0.3	>100
3	PEA-6T	Air	25	5	55	7.9 ± 0.4	>100
4	PEA-6T	Water	25	6	60	7.9 ± 0.3	>100
			30	6	55	7.6 ± 0.2	>100
			40	5.5	51	7.6 ± 0.3	>100
			50	4	46	7.5 ± 0.5	~40
5	PEA-6T	Methanol	25	6	65	7.8 ± 0.2	>100
6	PEA-6T	Ethanol	25	6.5	73	7.8 ± 0.2	>100
7	PEA-6T	*N*-butanol	25	8	90	7.6 ± 0.2	~40
8	PEA-6T	(0.9 wt%) NaCl	25	5.5	55	7.6 ± 0.2	>100
			50	3.5	42	7.1 ± 0.6	~55
9	PEA-6T	(3.5 wt%) NaCl	25	6	65	7.7 ± 0.2	>100
			50	4	33	6.8 ± 0.7	~60

*^a^* Induced bending time of PEA-*n*T films was tested under ultraviolet (λ = 365 nm, 40 mW cm^−2^) and visible light irradiation (λ > 510 nm, 30 mW cm^−2^). *^b^* Bending amplitude was calculated according to its uniform cycle times, which are 40 times for 50 °C water, 40 times for *N*-butanol, 40 times for 0.9 wt% NaCl, and 60 times for 3.5 wt% NaCl. *^c^* Cycling number was calculated according to its uniform cycle times. If the cycle exceeds 100 times, the results of 100 times will be used for calculation >100.

## Data Availability

No new data were created or analyzed in this study. Data sharing is not applicable to this article.

## Supplementary Materials

The following supporting information can be downloaded at: https://www.mdpi.com/article/10.3390/molecules30102106/s1, Scheme S1: Chemical structure and synthetic procedure of M-EA; Figure S1: POM images of PEA-*n*T (*n* = 2, 4, 6): (a) PEA-2T upon cooling to 133 °C and annealing for 30 min (during the first cooling process); (b) PEA-4T upon cooling to 112 °C and annealing for 30 min (during the first cooling process); (**c**) PEA-6T upon cooling to 97 °C and then annealed for 30 min (during the first cooling process). The scale bar is 20 μm; Figure S2: UV and visible light-induced photoisomerization cycles of the PEA-6T thin film at 25 °C. (In each cycle, the ultraviolet irradiation time is 65 s and the visible light irradiation time is 110 s); Figure S3: Photoresponsivity of the thin PEA-2T film in the UV (a) and visible light (b) at 25 °C; Figure S4: Photoresponsivity of the thin PEA-4T film in the UV (a) and visible light (b) at 25 °C; Figure S5: Photographs of azo polymer films in POM, Unstretched (a1, b1, c1) and stretched (a2, b2, c2) at room temperature; Photos of PEA-2T polymer films (a1, a2), PEA-4T polymer films (b1, b2), PEA-6T polymer films (c1, c2), at angles *θ* of 0° (left figure) and 45° (right figure) to the analyzer under orthogonal POM, with a scale size of 500 μm; Figure S6. SAXS of the Unstretched PEA-2T-2 film (a1), PEA-4T film (b1), and PEA-6T film (c1) and the uniaxially oriented PEA-2T film (a2), PEA-4T film (b2), and PEA-6T film (c2) (a strain of 235%); Figure S7: SAXS of integration on the uniaxially oriented PEA-2T film, PEA-4T film, and PEA-6T film (a strain of 235%); Figure S8: Photo of photo-induced bending/non-bending of azobenzene polymer PEA-2T stretch film under ultraviolet light (365 nm, 40 mW cm^−2^) and visible light (>510 nm, 30 mW cm^−2^) irradiation at room temperature; Figure S9: Photo of photo-induced bending/non-bending of azobenzene polymer PEA-4T stretch film under ultraviolet light (365 nm, 40 mW cm^−2^) and visible light (>510 nm, 30 mW cm^−2^) irradiation at room temperature; Figure S10: Three-dimensional shape reprogramming of uniaxially oriented PEA-6T thin films and the optical deformation behavior of the resulting reshaped optical actuators. (a) Photo of polymer film obtained by solution casting method, length ∗ width ∗ thickness: 11 mm ∗ 4 mm ∗ 64 μm; (b) the uniaxially oriented PEA-6T film (235% strain, 37 mm ∗ 2 mm ∗ 37 μm; (c) reshape film with one end containing approximately 1 mm of unstretched film) into a helical band at 26 °C; (d) place it at 26 or 80 °C for 20 days (or at 100 °C for 2 days); (e–i) the optical deformation behavior of helical bands at room temperature under irradiation of ultraviolet light (365 nm, 40 mW cm^−2^) (e–f) and visible light (λ > 510 nm, 30 mW cm^−2^) (h–i). The scale in the figure is 10 mm; Figure S11: The recycling process of light-deformed PEA-6T film. Firstly, dissolve the chopped PEA-6T film in hexafluoro isopropanol, pour the solution into a PTFE mold, evaporate the solvent, cut the peeled film into strips, and then stretch it to 235% strain (all processes are carried out at room temperature). The photo deformation properties of reprocessed PEA-6T film strips (10 mm ∗ 1 mm ∗ 37 μm) under ultraviolet light (365 nm, 40 mW cm^−2^) and visible light (λ > 510 nm, 30 mW cm^−2^). The scale in the picture is 10 mm; Figure S12: 1H NMR spectra of PEA-6T and recovered polymer; Figure S13: Contact angles under different solutions at 25 °C; Table S1: Contact angles under different solutions; Figure S14: FTIR of the uniaxially orientated PEA-6T films in air and water at 25 °C; Table S2: Brightness Level of POM in different solvents in different soaking times; Figure S15: (a) For UV dynamic overlay every 0.5 s, (b) Visible light dynamic overlay every 5 s. This includes air (a1, b1), water (a2, b2), methanol (a3, b3), ethanol (a4, b4), 0.9 wt% (a5, b5) and 3.5 wt% (a6, b6) sodium chloride aqueous solutions under ultraviolet light (365 nm, 40 mW cm^−2^) and visible light (λ > 510 nm, 30 mW cm^−2^); Table S3: The data of uniaxially oriented PEA-6T film (a strain of 235%); Figure S16: Photos of uniaxially orientated films in light deformation with different solvents in different soaking times. (a) Water (b) 0.9 wt% NaCl (c) 3.5 wt% NaCl (d) methanol (e) ethanol (f) *N*-butanol at 25 °C; Figure S17: Comparison diagram of polymer film swelling to different degrees after soaking in solvent for 30 days. The red dotted line is the length of the uniaxially oriented film soaked in water for 30 days, and the red arrow points to the swelling direction. POM in different solvents with different soaking times. (a) Water (b) 0.9 wt% NaCl (c) 3.5 wt% NaCl (d) methanol (e) ethanol (f) *N*-butanol at 25 °C. The swelling rate is determined by the film width after the film (except water) is soaked for 30 days, and the film width after the film is soaked in water for 30 days. Figure S18: FTIR of the uniaxially orientated PEA-6 T films in methanol, ethanol, and butanol at 25 °C; Table S4: Comparison of the photodeformation behaviors of the azo films [57,58]. Figure S19. Two different angle testing methods.
