# Supplementary material for: Study on Photodeformation of Solvent Resistance in Hydrogen-Bonded Cross-Linked Main-Chain Azobenzene Films
Source: Molecules. 2025 May 9;30(10):2106. doi: 10.3390/molecules30102106 (PMC12113695; doi:10.3390/molecules30102106)
Supplement: Supplementary file 1 [file molecules-30-02106-s001.zip › molecules-3579869-supplementary.pdf]

Supporting Information

Study on Photodeformation of Solvent Resistance in Hydrogen-bonded Cross-linked Main-chain Azobenzene Films

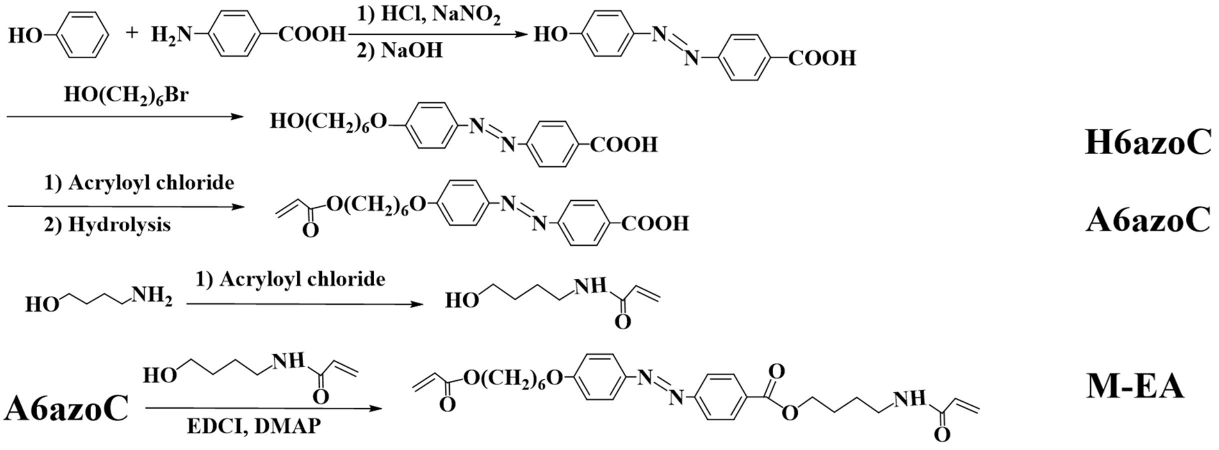

Scheme S1. Chemical structure and synthetic procedure of M-EA

POM of the main-chain azobenzene polymers

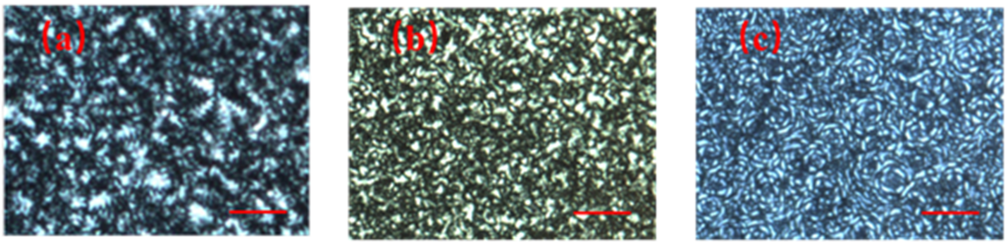

Figure S1. POM images of PEA-*n*T (*n* =2, 4, 6): (a) PEA-2T upon cooling to 133°C and annealing for 30 min (during the first cooling process); (b) PEA-4T upon cooling to 112°C and annealing for 30 min (during the first cooling process); (c) PEA-6T upon cooling to 97°C and then annealed for 30 min (during the first cooling process). The scale bar is 20 μm.

The thin PEA-nT films for the photoresponsivity study

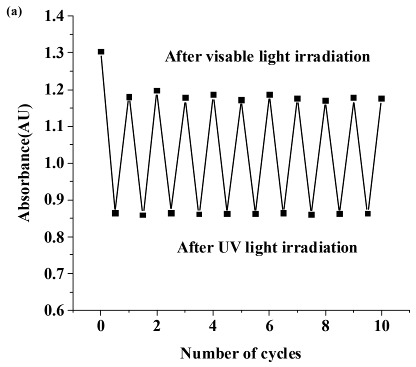

Figure S2. UV and visible light-induced photoisomerization cycles of the PEA-6T

thin film at 25 °C. (in each cycle, the ultraviolet irradiation time is 65 s and the visible light irradiation time is 110s).

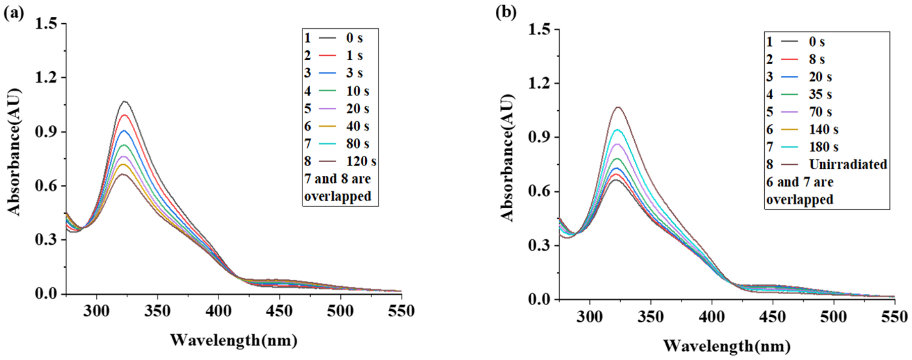

Figure S3. Photoresponsivity of the thin PEA-2T film in the UV (a) and visible light (b) at 25 °C

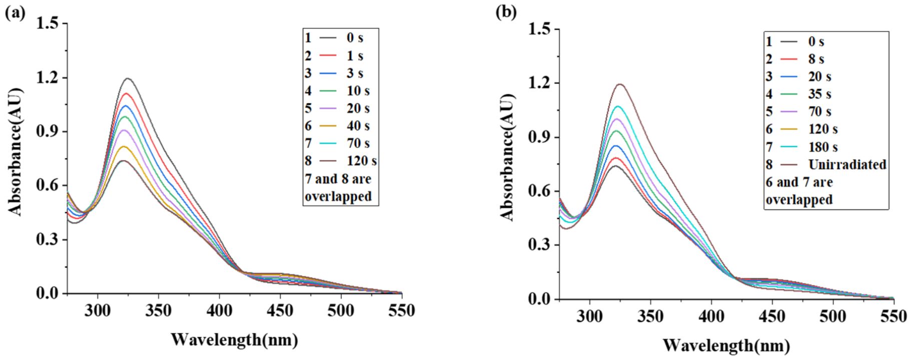

Figure S4. Photoresponsivity of the thin PEA-4T film in the UV (a) and visible light (b) at 25 °C

POM of the uniaxially oriented PEA-nT films

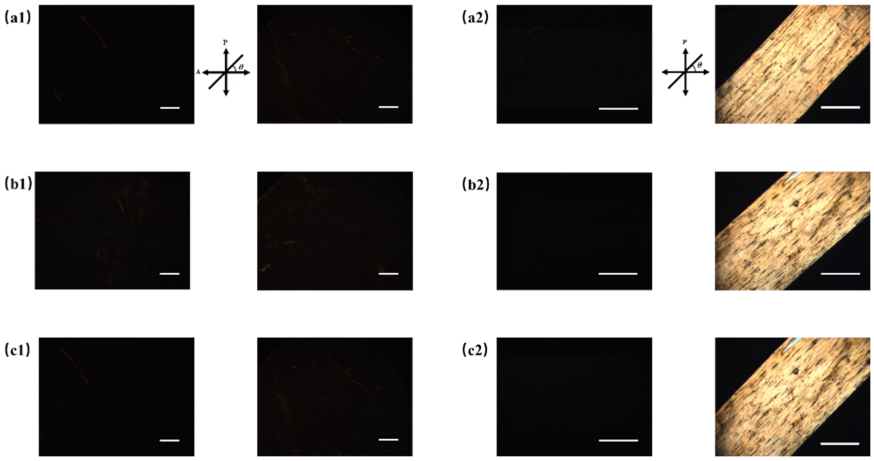

Figure S5. Photographs of azo polymer films in POM, Unstretched (a1, b1, c1) and stretched (a2, b2, c2) at room temperature; Photos of PEA-2T polymer films (a1, a2), PEA-4T polymer films (b1, b2), PEA-6T polymer films (c1, c2),at angles θ of 0 ° (left

figure) and 45 ° (right figure) to the analyzer under orthogonal POM, with a scale size of 500 μm.

Determination of degree of orientation in the uniaxially oriented PEA-nT films

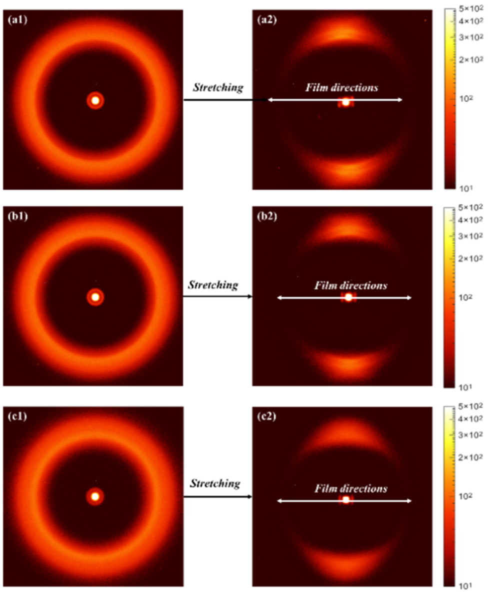

Figure S6. SAXS of the Unstretched PEA-2T-2 film (a1), PEA-4T film (b1) and PEA-6T film (c1) and the uniaxially oriented PEA-2T film (a2), PEA-4T film (b2) and PEA-6T film (c2) (a strain of 235%)

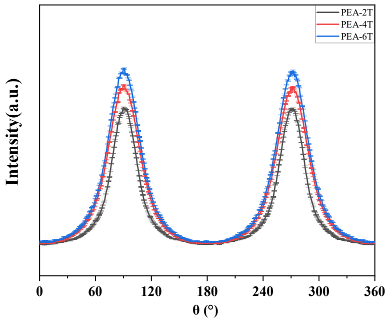

Figure S7. SAXS of integration on the uniaxially oriented PEA-2T film, PEA-4T film and PEA-6T film (a strain of 235%)

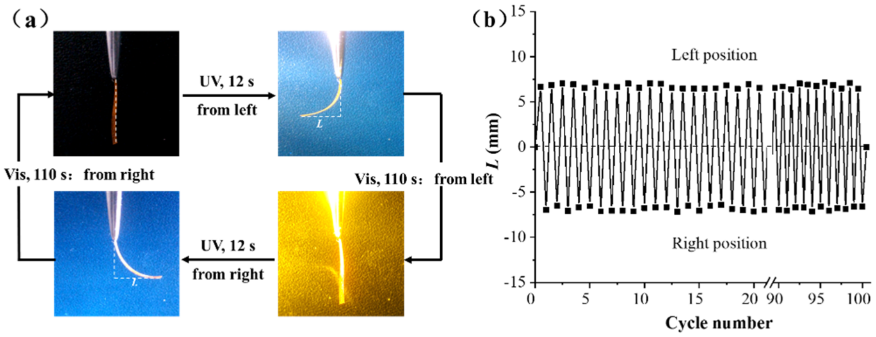

Figure S8. Photo of photo induced bending/non bending of azobenzene polymer PEA-2T stretch film under 365 nm ultraviolet light (40 mWcm^{-2}) and visible light (>510 nm, 30 mWcm^{-2}) irradiation at room temperature

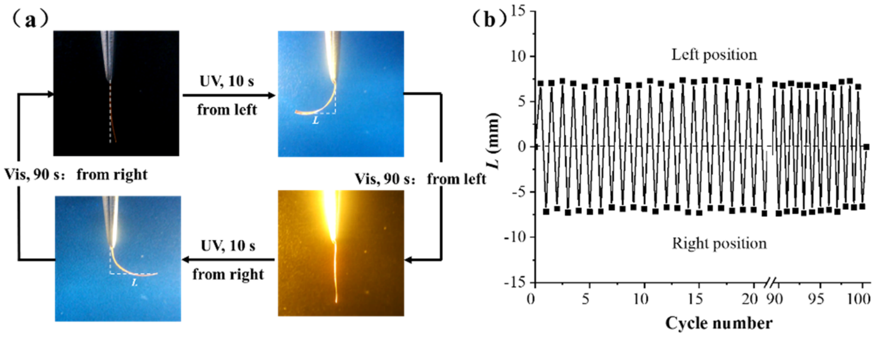

Figure S9. Photo of photo induced bending/non bending of azobenzene polymer PEA-4T stretch film under 365 nm ultraviolet light (40 mWcm^{-2}) and visible light (>510 nm, 30 mWcm^{-2}) irradiation at room temperature

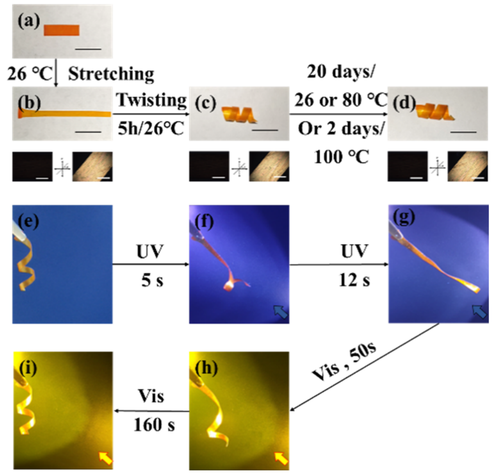

Figure S10. 3D shape reprogramming of uniaxially oriented PEA-6T thin films and the optical deformation behavior of the resulting reshaped optical actuators. (a) Photo of polymer film obtained by solution casting method, length * width * thickness: 11 mm *4 mm * 64 μm; (b) the uniaxially oriented PEA-6T film (235% strain, 37 mm * 2 mm

* 37 μm; (c)Reshape film with one end containing approximately 1 mm of unstretched film) into a helical band at 26 °C; (d) place it at 26 or 80 °C for 20 days (or at 100 °C for 2 days); (e-i) The optical deformation behavior of helical bands at room temperature under irradiation of ultraviolet light (365 nm, 40 mW cm^{-2}) (e-f) and visible light (λ>510 nm, 30 $mWcm^{-2}$) (h-i). The scale in the figure is 1 cm

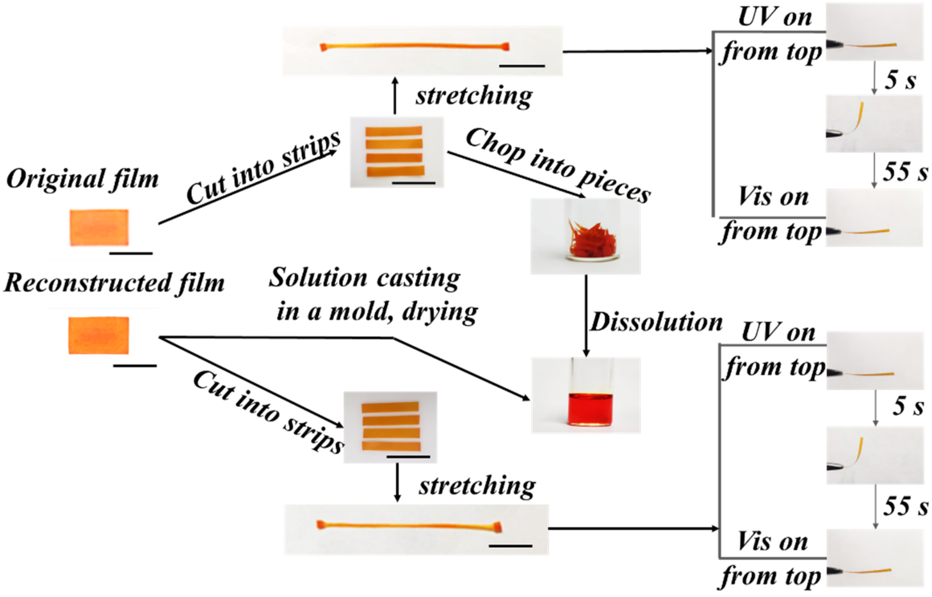

Figure S11. The recycling process of light deformed PEA-6T film. Firstly, dissolve the chopped PEA-6T film in hexafluoro isopropanol, pour the solution into a PTFE mold, evaporate the solvent, cut the peeled film into strips, and then stretch it to 235% strain (all processes are carried out at room temperature). The optical deformation properties of reprocessed PEA-6T film strips (10 mm * 1 mm * 37 μm) under ultraviolet light (365 nm, 40 $mWcm^{-2}$) and visible light (λ>510 nm, 30 mW cm^{-2}). The scale in the picture is 1 cm.

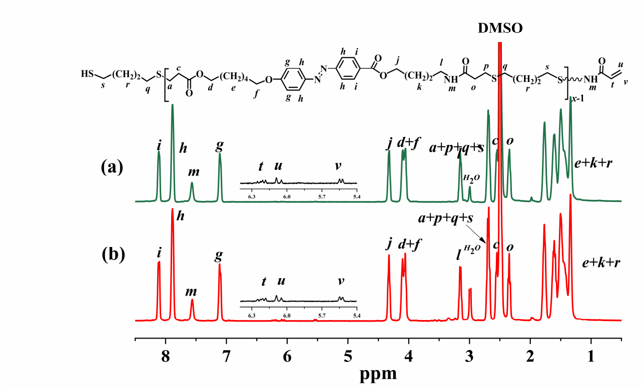

Figure S12. ^{1}H NMR spectra of PEA-6T and recovered polymer

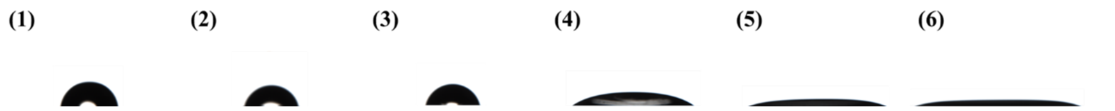

Figure S13. Contact angles under different solutions at 25 °C

Contact angles under different solutions (25 °C), where (1) is pure water; (2) 0.9wt.% NaCl aqueous solution; (3) 3.5wt.% NaCl aqueous solution; (4) Methanol solution; (5) Ethanol solution; (6) *N*-butanol solution;

Table S1. Contact angles under different solutions

Solvent	Contact angles
Water	82.3 ± 1.6
0.9 wt% NaCl	73.6 ± 1.2
3.5 wt% NaCl	77.5 ± 1.3
Methanol	28.0 ± 0.5
Ethanol	24.0 ± 1.5
N-Butanol	17.8 ± 1.1

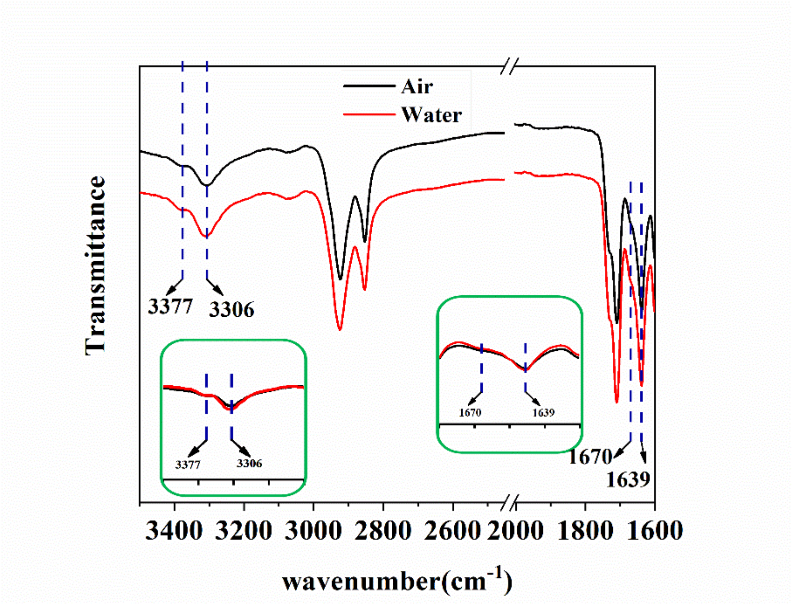

Figure S14. FTIR of the uniaxially orientated PEA- 6T films in air and water at 25 °C

Table S2. Brightness Level of POM in different solvents in different soaking time

	Water[a]	Water[b]	Water[c]	Water[d]
I[e]	169.541	163.735	169.716	168.916
II[e]	175.591	175.343	169.203	165.203
III[e]	172.166	171.214	176.325	175.325
Average[f]	172.4±3.0	170.1±5.9	171.7±4.0	169.8±5.1
	0.9 wt% NaCl[a]	0.9 wt% NaCl[b]	0.9 wt% NaCl[c]	0.9 wt% NaCl[d]
I[e]	153.436	152.695	150.459	149.881
II[e]	163.818	167.025	168.127	162.352
III[e]	171.532	164.516	160.315	165.924
Average[f]	162.9±9.1	161.4±7.7	159.8±8.6	159.1±7.3
	3.5 wt% NaCl[a]	3.5 wt% NaCl[b]	3.5 wt% NaCl[c]	3.5 wt% NaCl[d]
I[e]	141.385	132.713	132.048	147.577
II[e]	145.069	142.231	146.941	140.524
III[e]	133.274	147.984	139.520	134.305
Average[f]	139.9±6.0	141.0±7.7	139.5±7.4	140.8±6.6
	Ethanol[a]	Ethanol[b]	Ethanol[c]	Ethanol[d]
I[e]	182.378	158.806	139.404	123.048
II[e]	170.165	162.757	137.77	126.231
III[e]	165.58	173.667	152.511	136.859
Average[f]	170.1±8.7	165.1±7.7	143.2±8.1	128.7±7.2
	Methanol[a]	Methanol[b]	Methanol[c]	Methanol[d]
I[e]	177.499	165.214	168.848	143.477
II[e]	167.097	169.997	168.319	151.515
III[e]	165.712	159.718	155.032	156.469
Average[f]	172.7±6.4	165.0±5.1	158.1±7.8	150.5±6.6
	Butanol[a]	Butanol[b]	Butanol[c]	Butanol[d]
I[e]	118.053	97.986	71.267	56.364
II[e]	123.398	100.05	78.539	63.101
III[e]	120.407	106.277	75.239	58.144
Average[f]	120.6±2.7	101.4±4.3	75.0±3.6	59.2±3.5

[a] Preparation of polarizing brightness of Uniaxially oriented PEA-6T film (a strain of 235%) with dimensions of 10 mm * 1 mm * 36 um (length * width * thickness) was obtained by solution casting/mechanical stretching and soaked in solvent for 7 hours. [b] The only difference from "*a*" is that the soaking time is 7 days [c] The soaking time is 14 days. [d] The soaking time is 30 days. [e I, II, III] Use software Image J to select three pixels

with a pixel size of 6240 and calculate the average value. Set the minimum value to 0 and the maximum value to 255, with a brightness value of medium and a contrast value of medium.[f] Take the average of the three sets of brightness values obtained from "*e*"

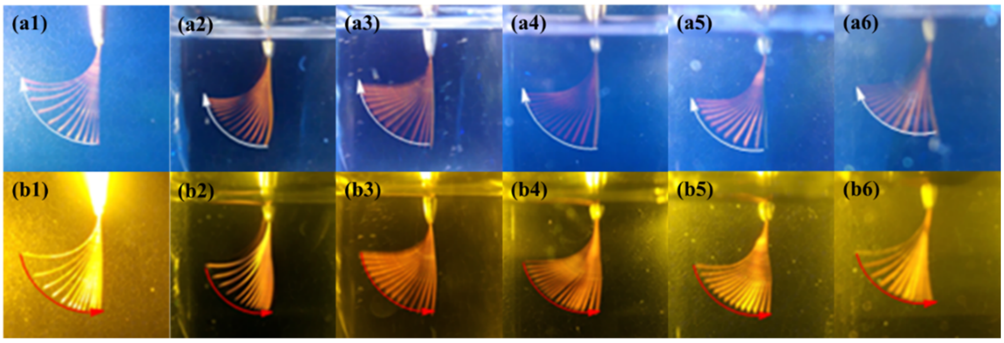

Figure S15. (a) For UV dynamic overlay every 0.5 seconds, (b) Visible light dynamic overlay every 5 seconds. This includes air (a1, b1), water (a2, b2), methanol (a3, b3), ethanol (a4, b4), 0.9 wt% (a5, b5) and 3.5 wt% (a6, b6) sodium chloride aqueous solutions under ultraviolet light (365 nm, 40 mWcm^{-2}) and visible light (λ>510 nm, 30 mW/cm^{-2});

Table S3. The data of Uniaxially oriented PEA-6T film (a strain of 235%)

	Water			0.9 wt% NaCl		
Time	Bending angle (°) [a]	Orientation (%) [b]	Photoinduced stress (kPa) [c]	Bending angle (°) [a]	Orientation (%) [b]	Photoinduced stress (kPa) [c]
7 hours	65.3±2.4	46.26	757.6±38.9	58.1±1.6	45.02	736.3±33.3
7 days	63.6±1.9	45.91	754.2±25.6	57.6±1.2	44.59	734.8±31.6
14 days	65.2±1.1	46.11	758.3±17.3	57.3±2.1	43.74	737.1±23.5
30 days	64.1±2.2	45.81	758.3±17.3	56.9±2.0	43.28	735.7±28.9
	3.5 wt% NaCl			Methanol		
Time	Bending angle (°) [a]	Orientation (%) [b]	Photoinduced stress (kPa) [c]	Bending angle (°) [a]	Orientation (%) [b]	Photoinduced stress (kPa) [c]
7 hours	56.1±2.2	42.03	691.5±22.3	55.6±1.2	45.41	648.3±27.7
7 days	54.5±1.5	41.77	705.7±18.7	55.2±0.9	43.74	639.9±17.8
14 days	53.8±2.1	41.66	707.0±18.7	54.9±1.1	42.03	629.9±20.8
30 days	53.3±0.9	40.98	703.2±21.5	53.8±2.4	39.70	610.1±28.0
	Ethanol			Butanol		
Time	Bending angle (°) [a]	Orientation (%) [b]	Photoinduced stress (kPa) [c]	Bending angle (°) [a]	Orientation (%) [b]	Photoinduced stress (kPa) [c]
7 hours	56.3±1.1	45.29	584.1±20.4	56.5 ± 1.9	41.77	403.7±15.6
7 days	53.5±1.4	41.77	521.5±19.1	53.2 ± 2.5	36.57	362.9±22.9
14 days	51.8±1.2	38.26	471.5±14.0	48.4 ± 3.2	29.15	327.1±14.7
30 days	47.2±1.6	36.57	421.5 ± 17.1	42.8 ± 2.9	20.47	242.3±32.8

[a] Maximum bending angle, [b] orientation of polymers [c] photoinduced stress in different solvent in different soaking time. (Soaking time for 7 hours means directly placing the uniaxially oriented polymer film into a solvent to test the results of 100 cycles, as shown in Table 3)

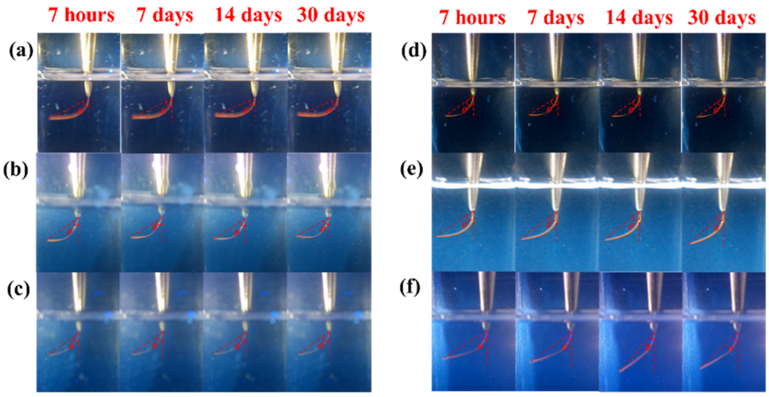

Figure S16. Photos of uniaxially orientated films of light deformation in different solvent in different soaking time. (a) water (b) 0.9 wt% NaCl (c) 3.5 wt% NaCl (d) Methanol (e) Ethanol (f) *N*-butanol at 25°C

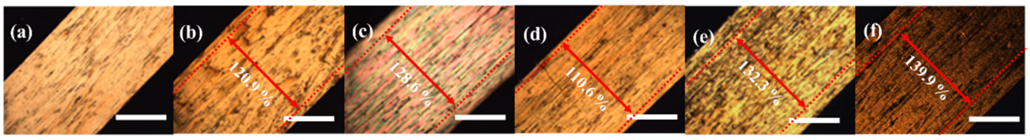

Figure S17. Comparison diagram of polymer film swelling to different degrees after soaking in solvent for 30 days. The red dotted line is the length of the uniaxially oriented film soaked in water for 30 days, and the red arrow points to the swelling direction. POM in different solvent in different soaking time. (a) water (b) 0.9 wt% NaCl (c) 3.5 wt% NaCl (d) Methanol (e) Ethanol (f) *N*-butanol at 25 °C. The swelling rate is determined by the film width after the film (except water) is soaked for 30 days and the film width after the film is soaked in water for 30 days.

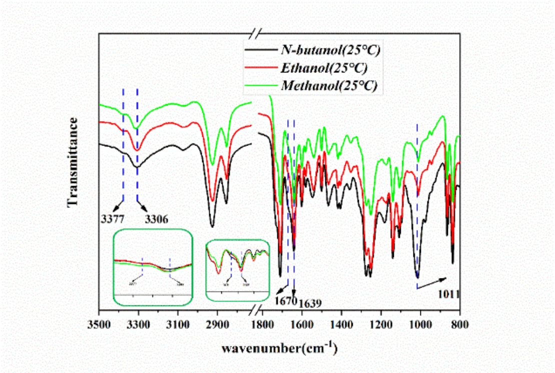

Figure S18. FTIR of the uniaxially orientated PEA- 6T films in Methanol, Ethanol and Butanol at 25°C

Table S4. Comparison of the Photodeformation Behaviors of the Azo films

Entry	Environment [a]	Actuator names [b]	Sample size [c]	T [e] (°C)	Bending angle (°) [f]	Light sources [i]	intensity (mW cm^{-2}) [j]	bending time (s) [k]	Bending/unbending Cycles [l]	Ref. [n]
1	Air	PEU-10 film	10 mm*1 mm*46 μm	25	-[g]	UV light (365 nm)	90	4.5	100	[42]
2	Air	LCP1 film	5 mm *0.5 mm*17 μm	25	-[g]	simulated sunlight	100	5	100	[57]
		LCP2 film	6mm*0.5mm*17 μm	25	-[g]	simulated sunlight	100	5	100	
3	Air	PEA-4 film	10 mm*1 mm*36 μm	25	95 [h1]	UV light (365 nm)	40	12	100	[25]
4	Air	PAZO/PU Blend Films	3 mm * 1 mm * 30 μm	80	144 [h1]	UV light (365 nm)	27	6	-[m]	[58]
5	Air	PEA/PDA@PI bilayer	10 mm * 1 mm *106 μm	25	35 [h2]	NIR light (808 nm)	0.88	1	100	[27]
	Air	PEA/PDA film	10 mm * 1 mm * 39 μm	25	60 [h2]	UV light (365 nm)	40	8	100	
	water	PEA/PDA film	10 mm * 1 mm * 39 μm	25	65 [h2]	UV light (365 nm)	40	9	100	
6	Air	DY-azo film	20 mm * 5 mm* - μm [d]	25	-[g]	UV light (365 nm)	170	2	-[m]	[43]
	Water			18	-[g]	UV light (365 nm)	170	30	-[m]	
7	Air	DAC 3AB@PI film	5 mm * 1mm * 20 μm	25	112 [h1]	UV light (365 nm)	70	8	50	[44]
	silicone oil	DAC 3AB@PI film	8 mm * 3mm * 20 μm.	80	45 [h2]	UV light (365 nm)	70	12	-[m]	
				100	22 [h1]			4	-[m]	
				120	5 [h1]			4	-[m]	
8	Air	PEA-6T	10 mm*1 mm*37 μm	25	68 [h2]	UV light (365 nm)	40	5	100	This
	water			25	65 [h2]			6	100	work
	water			40	55 [h2]			5.5	100	
	Methanol			25	56 [h2]			6	100	
	(0.9 wt%)			25	58 [h2]			5.5	100	
	NaCl									

[a] Environmental conditions for testing polymer photodeformation in different references. [b] The name of photo induced deformation film in different references. [c] The film size: length * width * thickness. [d] This paper has not yet mentioned its thickness. [e] Corresponding temperature of the environment in testing photodeformation. [f] The biggest bending. [g] The maximum bending angle has not been mentioned yet. [h] Two different angle testing methods. [i] Corresponding temperature of the environment during the testing process. [i] Different types of light sources. [j] Different light intensities of different light sources. [k] Maximum bending time. [l] Bending/unbending Cycles times [m] It has not yet been mentioned whether multiple reversible cycles of light exposure can be achieved.

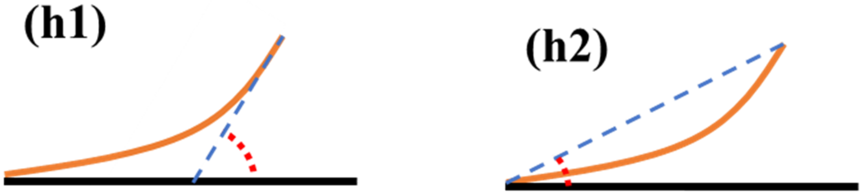

Figure S19. Two different angle testing methods.
